# Author Correction: An NF-κB-microRNA regulatory network tunes macrophage inflammatory responses

**DOI:** 10.1038/s41467-018-05720-5

**Published:** 2018-08-16

**Authors:** Mati Mann, Arnav Mehta, Jimmy L. Zhao, Kevin Lee, Georgi K. Marinov, Yvette Garcia-Flores, Li-Fan Lu, Alexander Y. Rudensky, David Baltimore

**Affiliations:** 1 Division of Biology and Biological Engineering, California Institute of Technology, Pasadena, CA 91125 USA; 2 David Geffen School of Medicine, University of California, Los Angeles, CA 90095 USA; 3 Department of Medicine, New York Presbyterian Hospital, Weill Cornell Medical College, 525 E 68th Street, New York, NY 10065 USA; 4 Division of Hematology Oncology, Memorial Sloan Kettering Cancer Center, 1275 York Avenue, New York, NY 10065 USA; 5 Division of Biological Sciences, University of California, San Diego, La Jolla, CA 92093 USA; 6 Moores Cancer Center, University of California, San Diego, La Jolla, CA 92093 USA; 7 Howard Hughes Medical Institute and Immunology Program, Ludwig Center at Memorial Sloan-Kettering Cancer Center, Ludwig Center at Memorial Sloan-Kettering Cancer Center, New York, NY 10065 USA

Correction to: Nature Communications 10.1038/s41467-017-00972-z, published online 11 Oct 2017.

Li-Fan Lu and Alexander Y. Rudensky, who supplied miR-146a floxed mice used in this study, were inadvertently omitted from the author list in the originally published version of this Article. This has now been corrected in both the PDF and HTML versions of the Article. The generation of the floxed mice has been described in detail by Cho and Lee et al^1^.
