# Supplementary material for: An NF-κB-microRNA regulatory network tunes macrophage inflammatory responses
Source: Nat Commun. 2017 Oct 11;8:851. doi: 10.1038/s41467-017-00972-z (PMC5636846; doi:10.1038/s41467-017-00972-z)
Supplement: Supplementary file 1 — Supplementary Information [file 41467_2017_972_MOESM1_ESM.pdf]

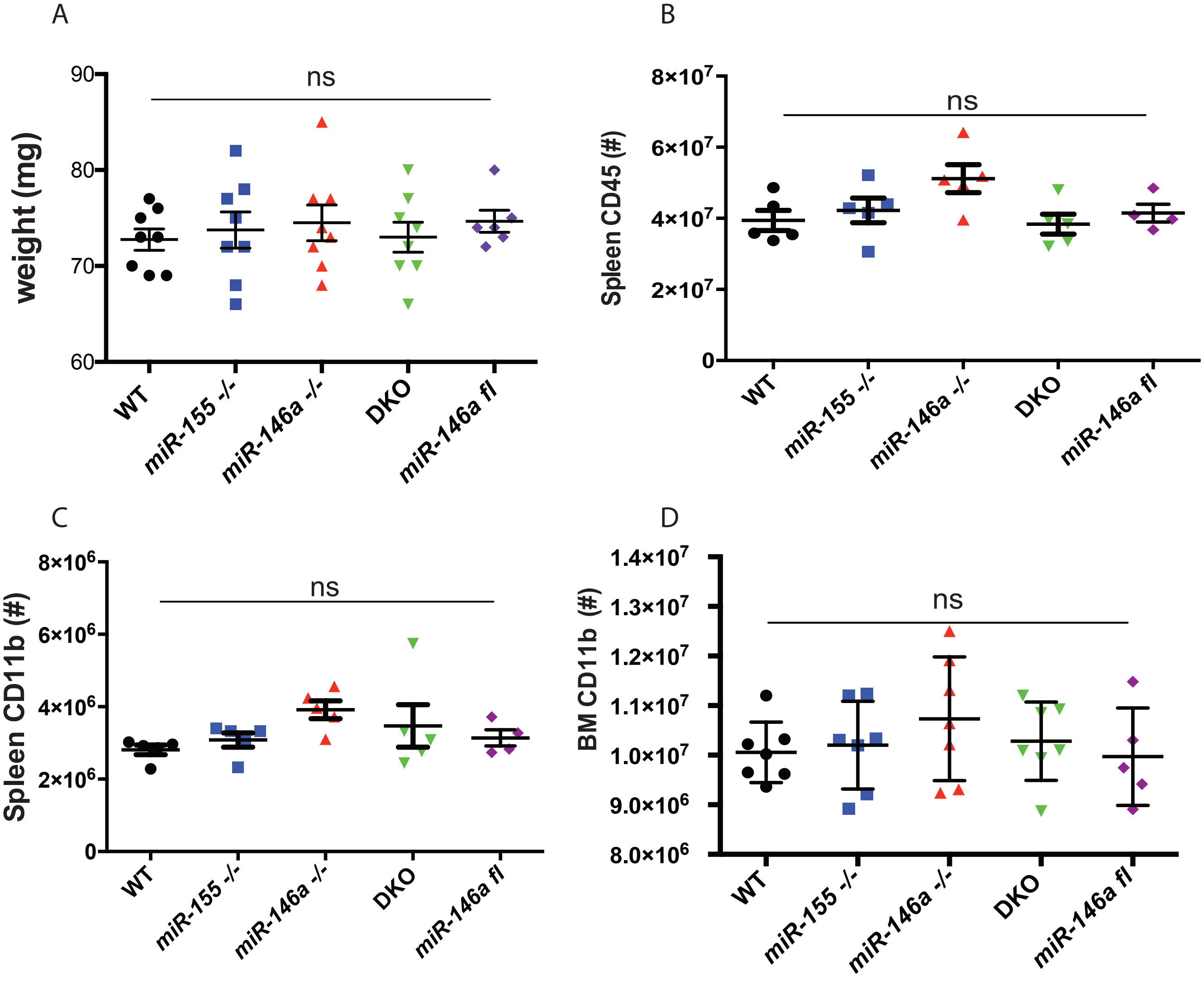

Supplementary Fig. 1. miR-146a does not lead to myeloproliferation in young mice. 20 weeks old WT, *miR-155-/-*, *miR-146a-/-*, double knock out (DKO) and *LyzM-Cre miR-146afl/fl* mice were analyzed for Spleen weight (A) spleen CD45^{+} cell numbers (B) spleen macrophages (C) as well as bone marrow macrophages numbers (D). N>4 (B, C)or N>5 (A,D) per group from at least two independent experiments, represented as mean ± SEM using 1 way ANOVA.

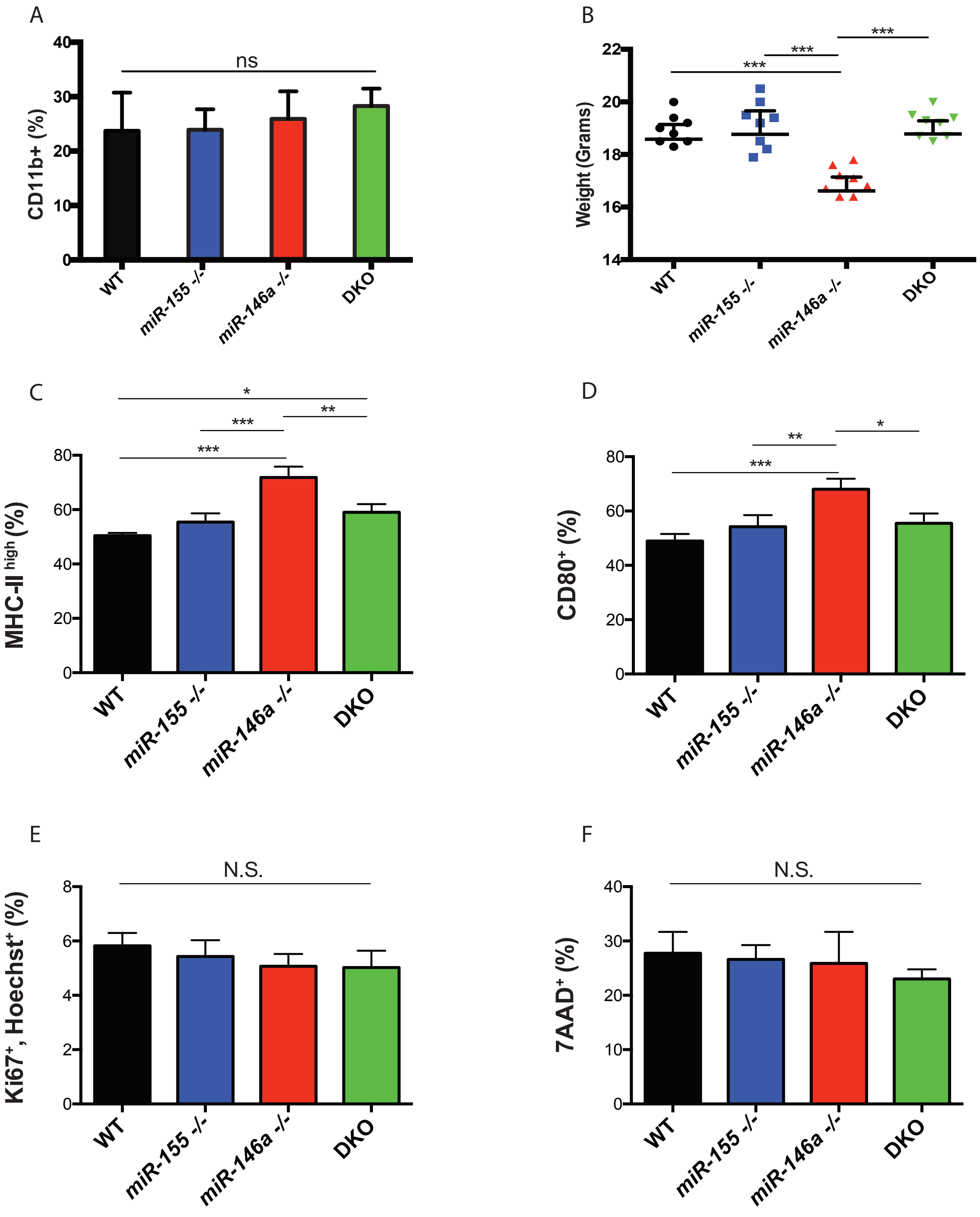

Supplementary Fig. 2. miR-155 expression is required for elevated acute inflammatory response in miR-146a deficient mice in response to *Listeria monocytogenes* and *Salmonella Typhimurium*.

CD11b macrophages levels (A), and body weight (B) were quantified 3 days after *Listeria monocytogenes* infection. Activation of BMMs from WT, *miR-155-/-, miR-146a-/-*, and double knock out (DKO) mice was quantified by the surface markers MHC-II (C) and CD80 (D). Cell proliferation (E), and death (F) was quantified using Ki67 + Hoechst, and 7AAD respectively. N>7(A,B) or N>6 (C-F) per group from 2 independent experiments, represented as mean ± SEM using 1 way ANOVA.

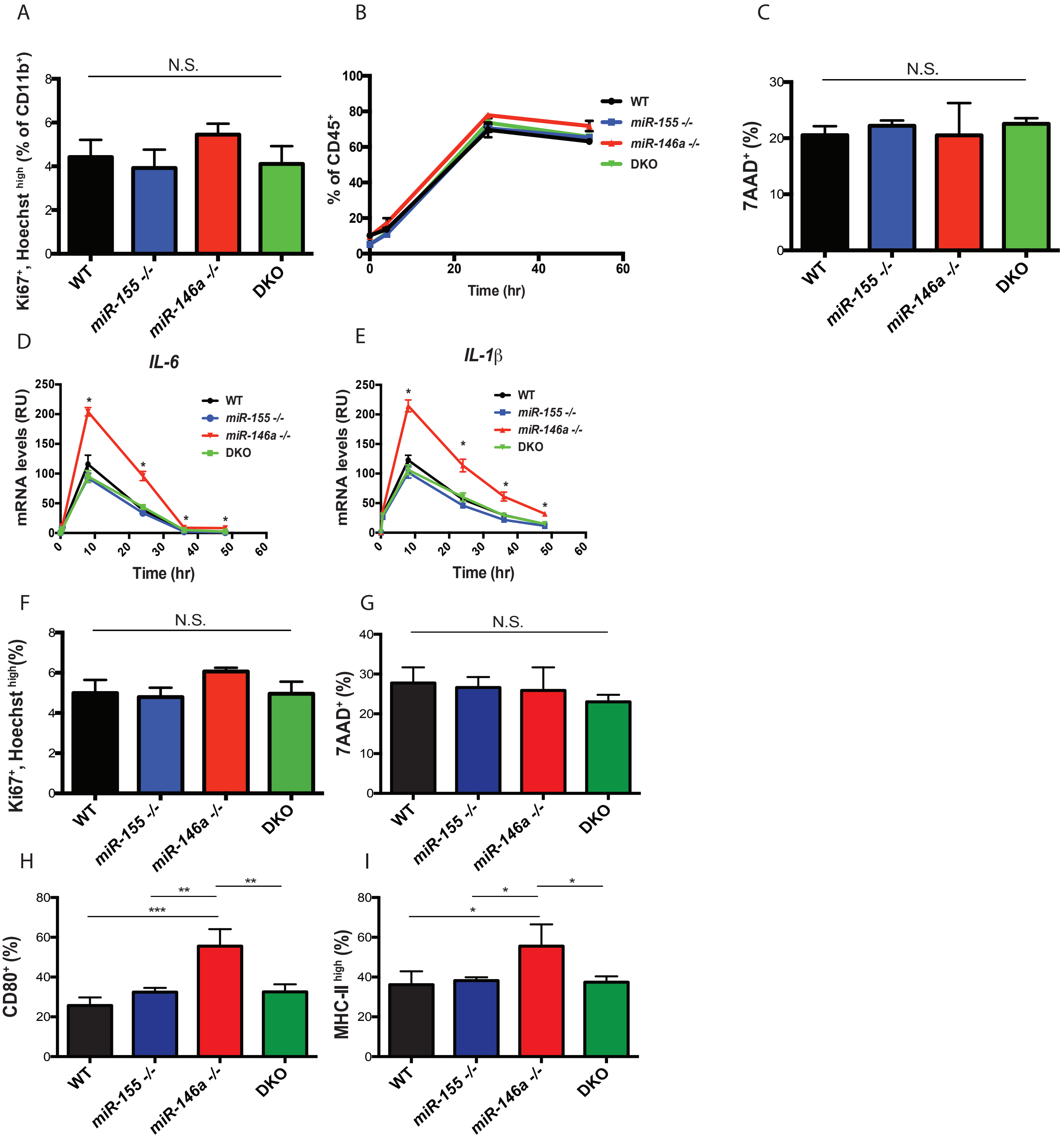

Supplementary Fig. 3. miR-155 expression is required for elevated acute inflammatory activation in *miR-146a-/-* BMMs. Periferal blood macrophage from WT, *miR-155-/-*, *miR-146a-/-*, and double knock out (DKO) mice were assayed for proliferation (A), frequency of total CD45 cells (B) and cell death (C) 48hrs after LPS stimulation. WT, *miR-155-/-*, *miR-146a-/-*, and DKO BMMs were stimulated with LPS for 48hrs. *IL-1β* (D) and *IL-6* (E) mRNA levels were quantified using qRT-PCR. Cell proliferation (F), cell death (G) and activation (H,I) were quantified using FACS. N>7 per group from two independent experiments and are represented as mean ± SEM. * denotes $p < 0.05$, ** denotes $p < 0.01$ and *** denotes $p < 0.001$using 1 way ANOVA (A,C,F-I) or 2 way ANOVA (B,D,E).

A

#	14050-MM_WT_0	14051-MM_WT_8	14052-MM_155_0	14053-MM_155_8	14054-MM_146_0	14055-MM_146_8	14056-MM_dko_0	14057-MM_dko_8
14050-MM_WT_0	1.00	0.82	1.00	0.82	0.99	0.80	0.99	0.82
14051-MM_WT_8	0.82	1.00	0.82	1.00	0.83	0.99	0.82	1.00
14052-MM_155_0	1.00	0.82	1.00	0.82	0.99	0.81	1.00	0.83
14053-MM_155_8	0.82	1.00	0.82	1.00	0.83	0.99	0.82	0.99
14054-MM_146_0	0.99	0.83	0.99	0.83	1.00	0.82	0.99	0.83
14055-MM_146_8	0.80	0.99	0.81	0.99	0.82	1.00	0.80	0.99
14056-MM_dko_0	0.99	0.82	1.00	0.82	0.99	0.80	1.00	0.82
14057-MM_dko_8	0.82	1.00	0.83	0.99	0.83	0.99	0.82	1.00

Pearson (log2(FPKM+1))

B

#	14050-MM_WT_0	14051-MM_WT_8	14052-MM_155_0	14053-MM_155_8	14054-MM_146_0	14055-MM_146_8	14056-MM_dko_0	14057-MM_dko_8
14050-MM_WT_0	1.00	0.82	1.00	0.82	0.99	0.81	0.99	0.82
14051-MM_WT_8	0.82	1.00	0.82	0.99	0.83	0.99	0.82	0.99
14052-MM_155_0	1.00	0.82	1.00	0.83	0.99	0.81	1.00	0.83
14053-MM_155_8	0.82	0.99	0.83	1.00	0.84	0.99	0.82	0.99
14054-MM_146_0	0.99	0.83	0.99	0.84	1.00	0.83	0.99	0.84
14055-MM_146_8	0.81	0.99	0.81	0.99	0.83	1.00	0.81	0.98
14056-MM_dko_0	0.99	0.82	1.00	0.82	0.99	0.81	1.00	0.83
14057-MM_dko_8	0.82	0.99	0.83	0.99	0.84	0.98	0.83	1.00

Spearman (log2(FPKM+1))

Supplementary Fig. 4. Gene expression profile similarity between WT, *miR-155-/-*, *miR-146a-/-*, and double knock out (DKO) bone marrow macrophages.

Pearson (A) and Spearman correlation (B) for all samples before (0), and 8hrs after (8) LPS stimulation.

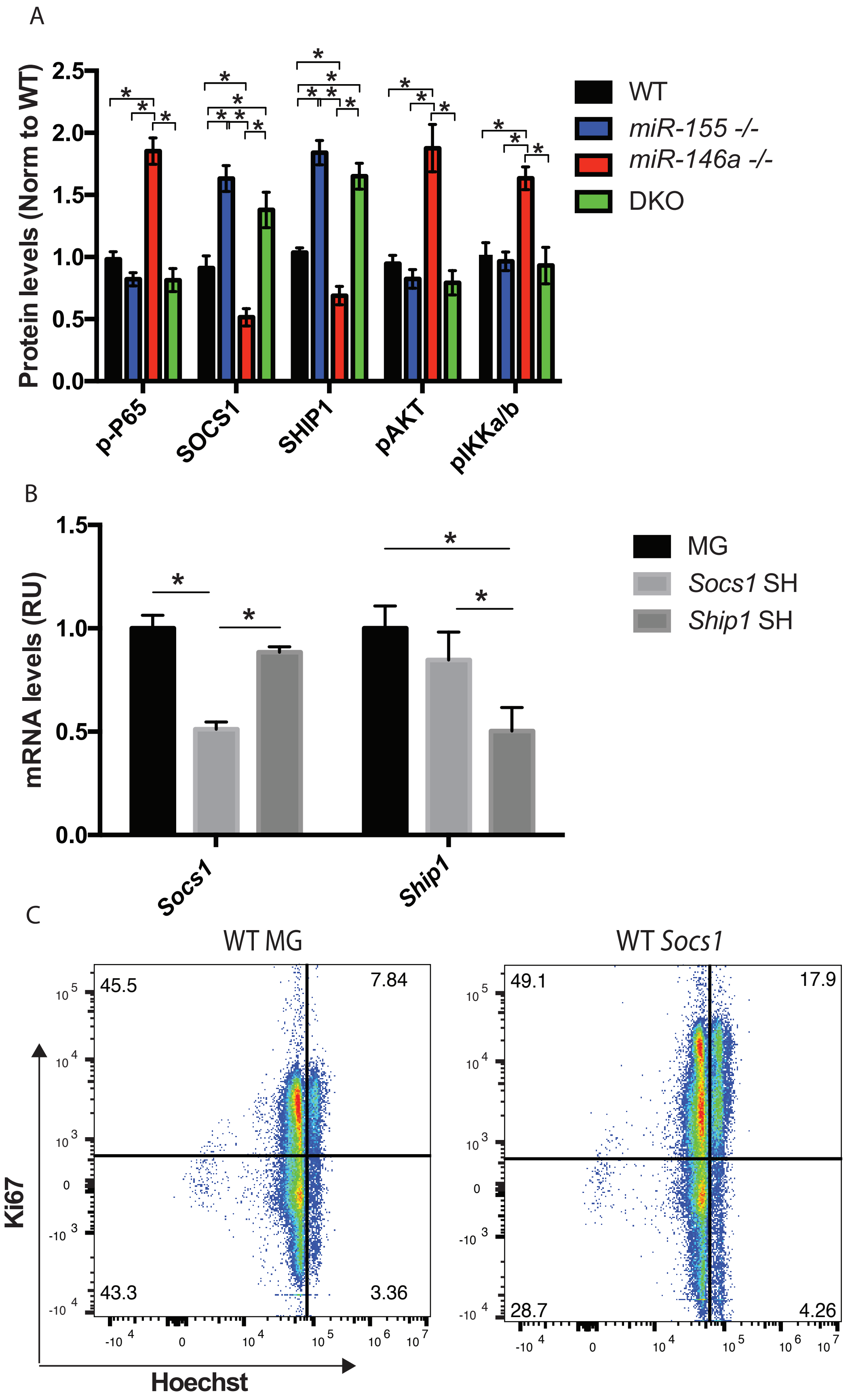

Supplementary Fig. 5. *Socs1* shRNA and *Ship1* shRNA leads to reduction in mRNA levels. (A) Protein expression quantification of Fig. 4D (B) RAW264.7 cells were infected with control (MG), *Socs1* shRNA, and *Ship1* shRNA expressing virus. 72hr post infection, cells were stimulated with LPS (100ng/ml) for 8hr prior to mRNA extraction. SHIP1 and SOCS1 levels were then quantified by qRT-PCR. (C) The proliferative state of WT CD11b^{+} cells transduced with control (WT MG), and *Socs1* shRNA (WT *Socs1*) was quantified using Hoechst and Ki67 staining. N=7 (A) N=4 (B) or N=8 (B) from two independent experiments.. (C,E,G,H) Presented mean ± SEM. * denotes $p < 0.05$, using 1 way ANOVA.

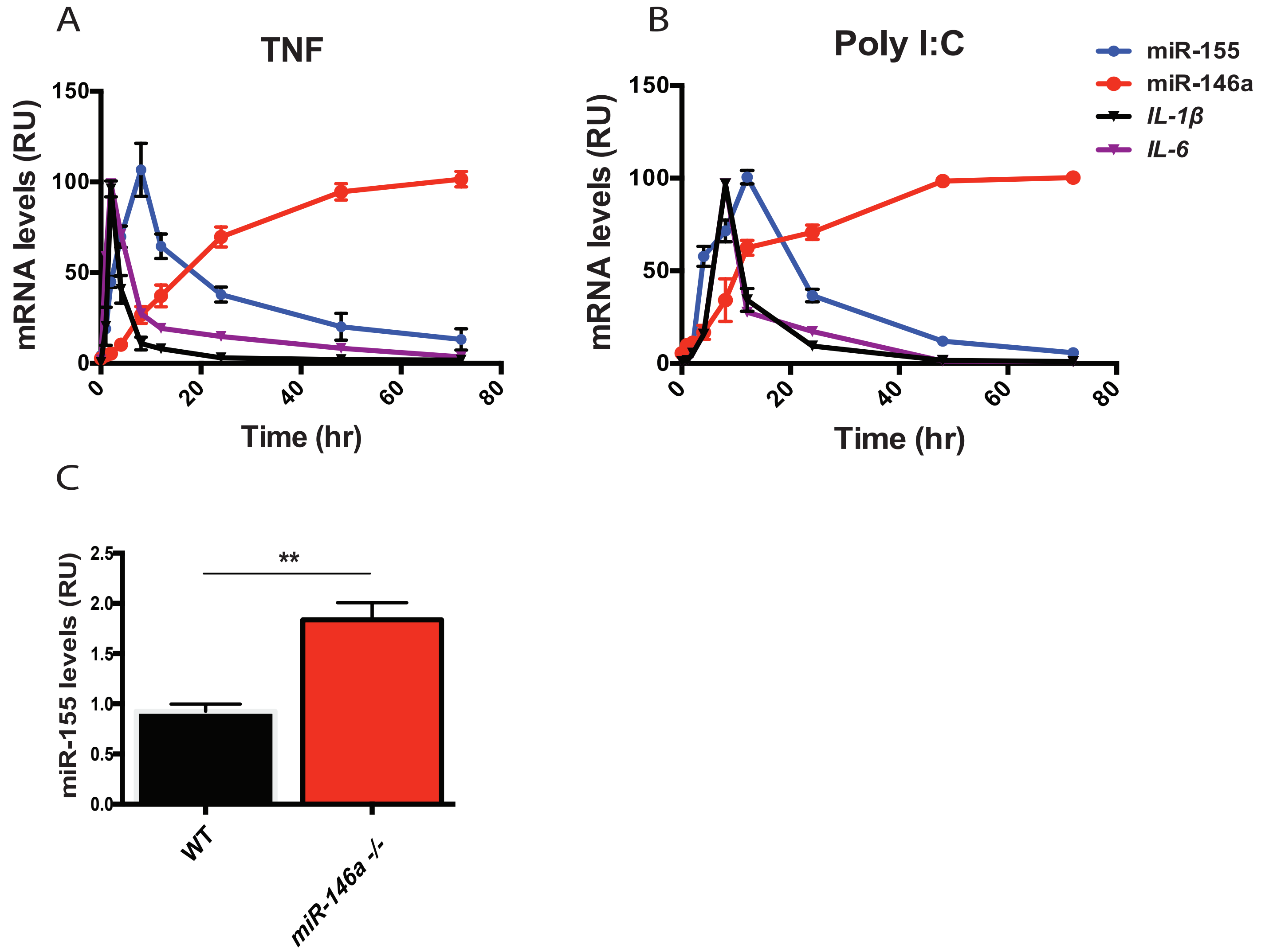

Supplementary Fig. 6. MiR-155 and miR-146a form a combined positive and negative auto regulatory loop to control precise NF-κB activity during inflammatory stimuli. The dynamic expression of miR-155, miR-146a, and NF-κB targets *IL-1β* and *IL-6* atdifferent time points after TNFa (A) and Poly I:C (B) stimulation. (C) miR-155 levels were quantified from unstimulated bone marrow macrophages of 6 months old WT and *miR-146a-/-* mice. N=4 from at least two independent experiments, represented as mean ± SEM. ** denotes p < 0.01, using Student's T test.

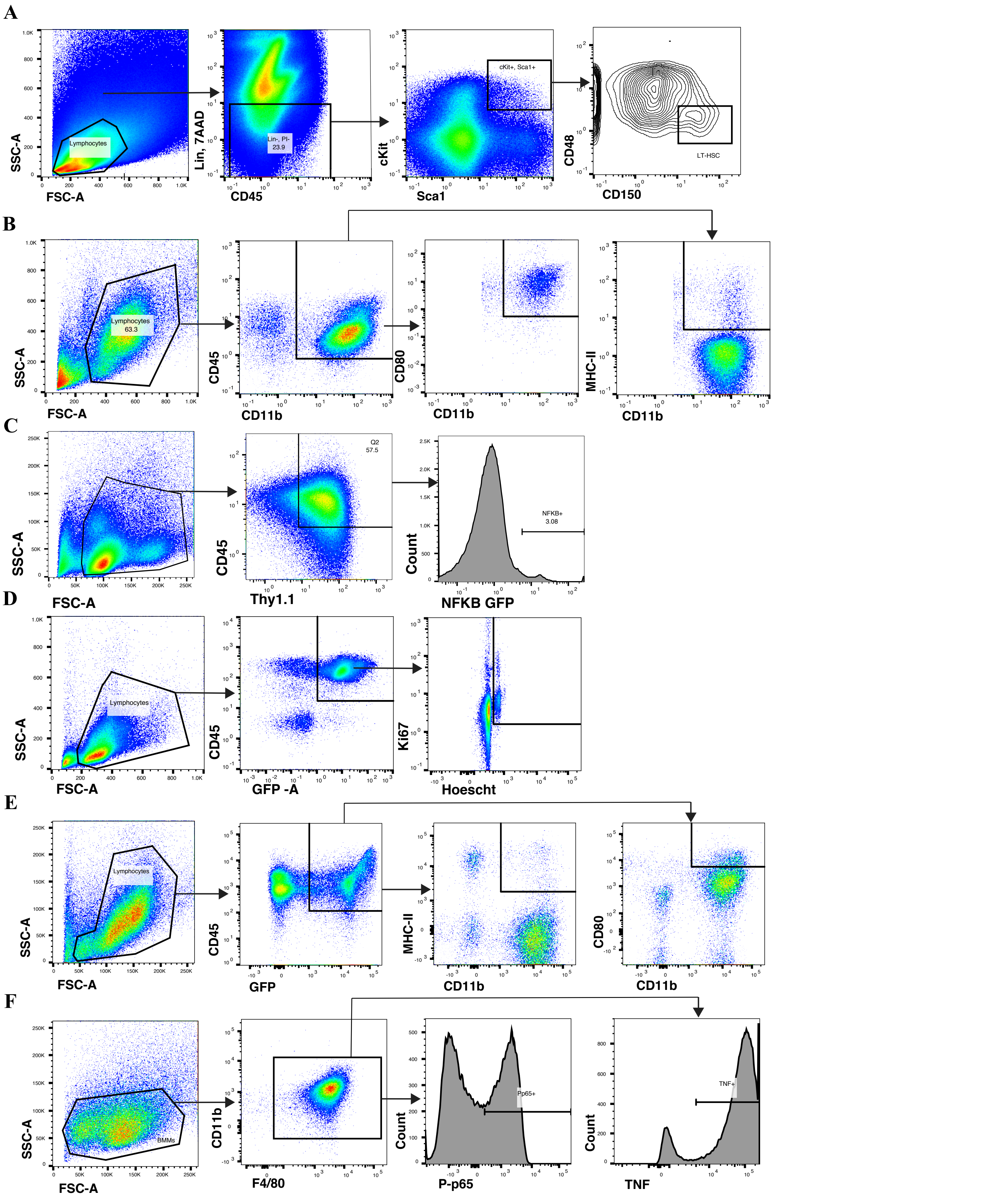

Supplementary Fig. 7 Gating strategy for FACS analysis for (A) HSCs as presented in Fig.1F, (B) MHC-II⁺ and CD80⁺ cells as presented in Fig 1D and 2E,F, (C) NFKB GFP as presented in Fig. 3C,D, (D) Proliferation markers as presented in Fig. 4F, (E) GFP⁺ MHC-II⁺, CD80⁺ cells as presented in Fig 5A-F, (F) P-p65 and TNF expression as presented in Fig. 6C.

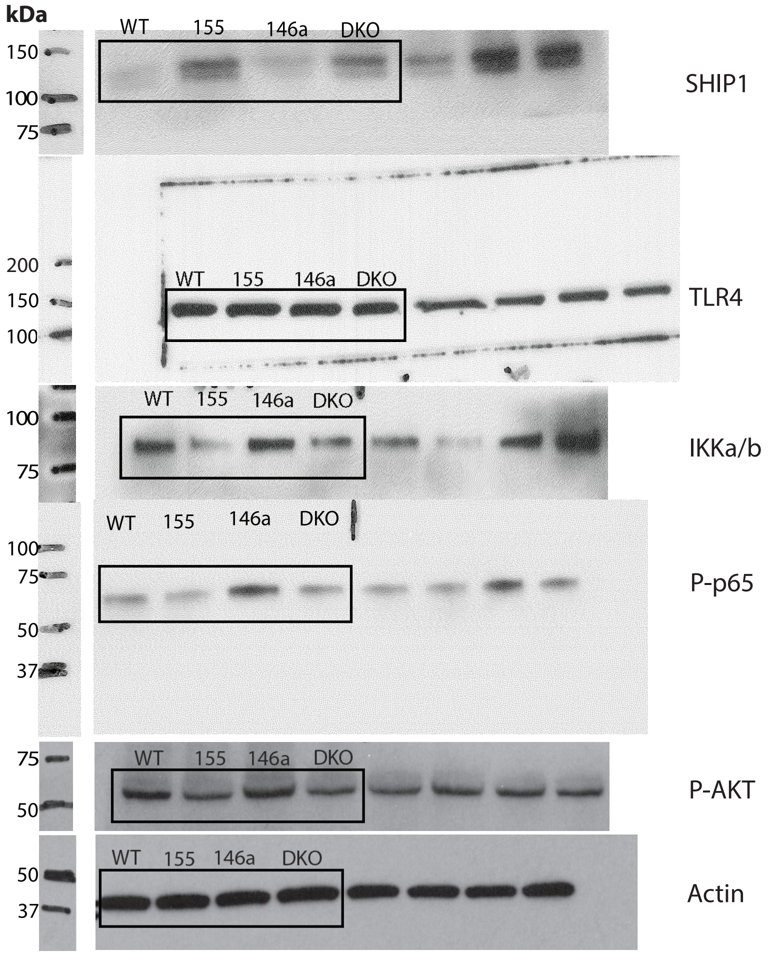

Supplementary Fig. 8. Uncropped images of immunoblots corresponding to Fig. 4D

Supplementary Table 1

MiRNA targets that are differentially expressed in KO BMMs based on RNA sequencing.

(Showing top genes for each miRNA)

miR-155 Differentially expressed targets	miR-146a Differentially expressed targets
S1pr1	Appl1
Zfp652	Arl5b
Ets1	Dcp1a
Zfp704	Ednrb
Mxi1	Irak1
Bach1	Lipa
Zfp703	Maff
Inadl	Prnp
Jarid2	Ptrh2
Jhdm1d	Stx3
Socs1	Traf6
Inpp5d	
Stard6	
Csnk1g2	
Fosl2	
Sfpi1	

Supplementary Table 2

A. Primers used for qRTPCR

PGK1:	For 5` TGCTGGAAAACCTCCGCTTTC 3`
	Rev 5` GGCTCGGAAAGCATCATTTT 3`
RL32	For – 5 AAGCGAAACTGGCGGAAAC 3
	Rev – 5 TAACCGATGTTGGGCATCAG 3
IL-6	For – 5 TAGTCCTTCCTACCCCAATTTCC 3
	Rev – 5 TTGGTCCTTAGCCACTCCTTC 3
TNFalpha	For – 5 GATCGGTCCCCAAAGGGATG 3
	Rev – 5 TGAGGGTCTGGGCCATAGAA 3
IL-1b	For – 5 GCA ACT GTT CCT GAA CTC AAC T 3
	Rev – 5 ATC TTT TGG GGT CCG TCA ACT 3
SHIP-1	For – 5 GCCCCTGCATGGGAAATCAA 3
	Rev – 5 TGGGTAGCTGGTCATAACTCC 3
SOCS1	For: 5’ CTCCTTGGGGTCTGTTGGC
	Rev: 5’ GCGTGCTACCATCCTACTCG
IRAK1	For – 5 CCAGAGGCAAAACTCCCAACA 3
	Rev – 5 AGAGCACCTCCCCAAATAGAG 3
TRAF6	For – 5 CCTGACGGTAAAGTGCCCAA 3
	Rev – 5 ACCTGGCACTTCTGGAAAGG 3
ETS1	For: CAAGCCGACTCTCACCATCA
	Rev: AGCTTTCAAGGCTTGGGACA
Bach1	For: GGAGTGAGTCACCTGACCG
	Rev: GGGAGAGCAGTGGAAAGCAT
IL-21	For: GGAGGAAAGAAACAGAAGCACAT
	Rev: CCGGACACAACATGGAAGTGAA
Bcl6	For: AAAGGCCGGACACCAGTTTT
	Rev: AACGTCCGTCAAGATGTCCC

B. Oligo sequences for overexpression and shRNA experiments

miR-146a overexpression sequence
gaaggctgtatgctgTGAGAACTGAATTCCATGGGTTGTTTTGGCCACTGACTGACAACCCATGATTCAGTTCTCAcaggacacaaggcctg
miR-155 overexpression sequence
gaaggctgtatgctgTTAATGCTAATTGTGATAGGGGTTTTGGCCACTGACTGACCCCTATCAATTAGCATTAAcaggacacaaggcctg
miR-146a and miR-155 overexpression sequence
CTGGAGGCTTGCTgaaggctgtatgctgTTAATGCTAATTGTGATAGGGGTTTTGGCCACTGACTGACCCCTATCAATTAGCATTAAcaggacacaaggcctgtaagaagttatgtattcatccaataattcaagccaagcaa gtatataggtgttttaatagttgtatgaaggctgtatgctgtgagaactgaattccatgggttgttttggccactgactgacaacccatgattcagttctcacaggacacaaggcctgTTACTCGCACTCACATGGAACAAATGGCCCA
SHIP1 shRNA
GAAGGCTGTATGCTGTATTACGTAATCGTGATAGGGGTTTTGGCCACTGACTGACCCCTATCAATTACGTAATACAGGACACAAGGCCTG
SOCS1 shRNA
GAAGGCTGTATGCTGAGTACCGGGTTAAGAGGGATGGTTTTGGCCACTGACTGACCATCCCTCAACCCGGTACTCAGGACACAAGGCCTG
