# Supplementary material for: An NF-κB-microRNA regulatory network tunes macrophage inflammatory responses
Source: Nat Commun. 2017 Oct 11;8:851. doi: 10.1038/s41467-017-00972-z (PMC5636846; doi:10.1038/s41467-017-00972-z)
Supplement: Supplementary file 3 — Description of Additional Supplementary Files [file 41467_2017_972_MOESM3_ESM.pdf]

Description of Additional Supplementary Files

File Name: Supplementary Data 1
Description: It contains a detailed GO enrichment list of molecular pathways with differential expression between WT, *miR-155-/-*, *miR-146a-/-* and DKO BMMs.
